# The Profession and the National Health Commissioners: Incapable of Work

**Published:** 1916-07-29

**Authors:** C. Tuckfield, R. W. Harris, Hugh Woods

**Affiliations:** Mr. A. Webb, 34/62.; General Secretary, London and Counties Medical Protection Society, Ltd., 32 Craven Street, Strand, W.C.


### THE EDITOR'S LETTER-BOX.

### The Profession and the National Health Commissioners.

### "Incapable of Work."

*To the Editor of* The Hospital.

Sir,—I am directed by the Council of the London and Counties Medical Protection Society, Ltd., to forward to you for publication the enclosed copy correspondence.

The majority of patients rightly certified for the purposes of the National Health Insurance sickness benefit cannot in the ordinary meaning of the words be stated to be "incapable of work," and it is therefore desirable that medical practitioners who wish to adhere strictly to verbal accuracy in their certificates should note the interpretation that will be placed upon the words "incapable of work" by the National Health Insurance Commission.

It is unfortunate that more suitable words were not inserted in the Act, but the next best thing is to have a definite statement by the National Health Insurance authorities stating what meaning they will attach to the words, and this appears clearly from the following correspondence.—I am, Sir, yours faithfully,

Hugh Woods, General Secretary.

32 Craven Street, Strand, London, W.C.,
June 27, 1916.

Note.—Dr. Fowler's qualification added was, "unfit, not incapable."

---

London and Counties Medical Protection Society, Ltd.,
32 Craven Street, Strand, London, W.C.
June 27, 1916.

Sir,—I am directed by the Council of this Society to call your attention to the enclosed letter received by a patient of Dr. C. Owen Fowler, a member of our Society, from the "National Deposit (Approved) Friendly Society" in reference to the enclosed medical certificates. Our Society strongly urges upon its members the necessity of adhering with the utmost strictness to truth and accuracy in medical certificates. Unfortunately, the wording of the certificate as approved by the National Health Insurance Commissioners is such that it is not true, in the ordinary meaning of the words, in a large number of cases where it is obviously proper and obviously intended that a medical certificate should be given.

Dr. Fowler, in the present instance, has, in the opinion of our Council, very properly added a remark obviating the false impression which would be given by the wording of the certificate. The patient in this case—as in a very large proportion of the cases which are rightly certified as "unfit for work"—could not properly be said to be "incapable of work," and Dr. Fowler, therefore, without altering the certificate, since that might be objected to, very properly added a remark defining the meaning which must in that case and in similar cases be attributed to the word "incapable," unless it ceases to be true.

Until the Commissioners, as we hope may be the case at an early date, decide to modify the wording of the required sickness certificate, it seems to be necessary for medical practitioners to add a note modifying the meaning of the word "incapable" when the patient—although in a condition which would make it wrong both in the interests of his own health and in the interests of the Friendly Society insuring him, to go to work, is yet quite capable of work if he is foolish enough to do it.

Our Council trusts that the Insurance Commissioners will in this case, and in any similar cases, take steps to prevent insured persons suffering because the doctor declines to give a certificate which is not strictly true, and to prevent pressure being put upon medical practitioners to make them sign certificates which they know to be untrue, and which they know would be regarded as untrue by a professional body such as the Council of this Society.—I am, Sir, yours faithfully,

(Sgd.) Hugh Woods, General Secretary.

The Secretary,
National Health Insurance Commission (England),
Buckingham Gate, S.W.

---

[*Copy.*]

National Deposit (Approved) Friendly Society,
37 Queen Square, Southampton Row, W.C.
June 16, 1916.

State Claim, 34/62/244.

Dear Sir,—I am in receipt of your letter of the 14th inst., together with communication which you have received from the above contributor, and two Forms Med. 40, which I return herewith. I would inform you that the medical practitioner should be advised that the Form Med. 40 was compiled by the Insurance Commissioners, and the certificates as amended cannot be accepted. Until medical certificates as required by the Commissioners' regulations are furnished, I regret that no benefit can be paid to the member. I enclose Form "P" in order that benefit may be paid on receipt of correct certificates.—Yours faithfully, (Sgd.) C. Tuckfield.

Mr. A. Webb, 34/62.

---

National Health Insurance Commission (England),
Buckingham Gate, London, S.W.,
July 10, 1916.

83247/16.

Sir,—In reply to your letter of the 27th ultimo, with reference to certificates given by Dr. C. Owen Fowler, I am directed by the National Health Insurance Commission (England) to state that the representations of your Council in the matter have been duly noted.

With regard to the wording of Form Med. 40, I am to point out that the phrase "incapable of work" is taken from Section 8 (*c*) of the National Insurance Act, 1911. The Commissioners have no power to give an interpretation of the phrase which will be binding on all parties. They desire me, however, to state that in any case of the kind referred to in your letter, in which the patient is in a condition which would make it wrong, both in the interests of his own health and in the interests of his Approved Society, for him to go to work, the doctor would, in the opinion of the Commissioners, be justified in giving a certificate of incapacity in the wording of Form Med. 40. Further, it would appear to the Commissioners that on a proper interpretation of the Medical Certification rules the doctor would, in such a case, be under an obligation to give a certificate on Form Med. 40.—I am, Sir, your obedient servant,

(Sgd.) R. W. Harris.

Hugh Woods, Esq., M.D., B.A., General Secretary,
London and Counties Medical Protection Society,
Ltd., 32 Craven Street, Strand, W.C.

